# A qualitative study of governance predicament on dengue prevention and control in Malaysia: the elite experience

**DOI:** 10.1186/s12889-021-10917-3

**Published:** 2021-05-06

**Authors:** Rosliza Abdul Manaf, Aidalina Mahmud, Anthony NTR, Siti Rohana Saad

**Affiliations:** 1 Health Management Unit, Department of Community Health, Faculty of Medicine and Health Sciences, Universiti Putra Malaysia, Serdang, Selangor Malaysia; 2 Hulu Selangor Health District Office, Kuala Kubu Bharu, Malaysia

**Keywords:** Good governance, Elite, District health office, Dengue prevention and control, Malaysia

## Abstract

**Background:**

The challenges faced by healthcare personnel in relation to dengue prevention and control are perennial but noticeably unexplored. It is often difficult to translate policies and decision making by the elite into astute management in consonance with the needs of rank-and-file personnel. In this study, we assess the impact of governance on dengue prevention and control activities in Malaysia as narrated by the elite.

**Methods:**

A qualitative study using a case-study approach was conducted between January 2019 and November 2019 in the districts of Gombak and Klang, where the relevant key informants were located. Nineteen interviews were conducted among elite healthcare personnel from different divisions: management, vector, laboratory, inspectorate, health promotion and entomology. Semi-structured interviews were conducted. The sample size was determined through saturation point criteria. Purposive sampling techniques were used to recruit the participants. The interviews were audio recorded, and the transcribed text was analysed with deductive thematic analysis.

**Results:**

Data analysis led to the development of 5 themes and 13 categories. The major principles of governance were embodied in a milieu of predicament, linked to constraints but also opportunities. The constraints resulted from inherent determinants of dengue outbreaks, the serviceability of governing policies and the macro-economics of budget allocation. The opportunities to sustain governance at the local operating level stem from a prevalent supportive internal management system, collaborative efforts among corresponding external government agencies and willingness to innovate and embrace novel technology.

**Conclusion:**

Elites are influential, often well-informed personnel tasked with making decisions that can reverberate across an organisation, impacting future plans and strategic policies. Political arrangements at higher levels will reflect in advance the tone of how governance in dengue prevention and control is operationalised by entities and individuals at lower levels of the health system. The prevailing centralised structure in the Malaysian health system will continue to entrench the position of the elite and intertwine it with governance and its predicaments.

**Supplementary Information:**

The online version contains supplementary material available at 10.1186/s12889-021-10917-3.

## Background

Governance matters, as it is concerned with how different stakeholders in the world function and operate as well as the rationale for each decision-making process [1]. The subject of governance has become essential and crucial for the public sector domain, especially in Malaysia. The prime minister of Malaysia has emphasised issues of governance, which has required the public sector to act within an operating model of good governance [2].

According to the Commission of Global Governance, there are four core principles of good governance [3]. First, good governance is the capacity of the state to work for the well-being of the people. Individuals employed in the public sector must be knowledgeable of state rules, regulations and policies to be of assistance to the government [4]. Having good knowledge of the relevant subject matter will facilitate a public servant’s capacity to operate effectively and efficiently [5]. Second, a transparent structural organisation led by accountable stewards will affect how well civil servants perform their roles in delivering services to the public [6, 7]. Third, it is essential that government-initiated and funded programmes be conducted with transparency and integrity. Finally, the rule of law, also an element of good governance, is closely associated with principles of performance and accountability [8]. Hence, the commission report confirms that good governance results from a number of intertwined elements that are and dependent on the corresponding principles [9].

The Malaysian government has named nine principles per the circulation by the chief secretary to the federal government [2, 10]. The first guideline stated the four principles of integrity, accountability, transparency and stewardship as moral rules for good governance in Malaysian government departments and agencies [11]. The next guideline in the same circular described another five principles of good governance for government departments and agencies: leadership, relationships of stakeholders, internal and external accountability, strategic management, performance monitoring and risk management. These guidelines were similar to the House of Governance Framework from the Australian National Audit Office (ANAO, 2003), which has seven principles [12]. The ANAO stated that stringent adherence to requirements for conformity and output, rather than pragmatism, are important benchmarks for attaining good governance in government organisations at all levels.

Governance in healthcare is increasingly being regarded as a salient theme in healthcare development and delivery. Previous reviews have highlighted the positive impact of good governance on the healthcare sector, directly or indirectly through its impact on income [13, 14]. The concept of good governance in the health system has been discussed in various domains, including mental health [15] and infectious diseases [16]. In fact, good governance is seen as a foundation for the strong and resilient health system that is required to manage disease outbreaks, including dengue fever.

The healthcare sector, specifically public health and the associated healthcare delivery system, is one context in which there is a scarcity of research based on interviews with elite healthcare personnel [17–19]. The term “elite” in social studies can be traced to the turn of the twentieth century, when those who were classified as elites and governed others were considered to possess superior capabilities [20]. By the mid-twentieth century, these clusters of governing segments had narrowed to a functional definition of “power elites” as legislators, judges, military officers, and prominent government office holders [21]. Recent definitions have suggested that elites are those who can use their attributes, behaviour, relationships, and privileged access to exercise power without significant challenge or defiance from those governed [22]. The nonconfrontational temperament that prevails among civil servants in Malaysian government sectors as a result of a deep-rooted feudalistic culture further adds to the predicament [23].

Malaysia’s healthcare system is a structured system under the responsibility of the Ministry of Health. Administratively, it consists of the central headquarters at the national level, state health departments and district health offices. Health policies are developed at the central level and passed down to the intermediate (state) and peripheral (district) levels for implementation.

Elites may wear different hats based on their expertise or talent as policy makers, inner-sanctum decision makers or simply division heads in the process of developing policies or building frameworks for rank-and-file personnel in the Ministry of Health [2, 24]. However, the operationalization of well-crafted policies or theoretical frameworks on dengue prevention and control often does not augur well for the capacities and capabilities at local district offices. In addition, there is no regulation to enforce policy implementation by healthcare personnel at all levels [25]. This study assessed the impact of governance on dengue prevention and control activities in Malaysia as narrated by the elite.

## Methods

### Study setting

**Fig. 1:**
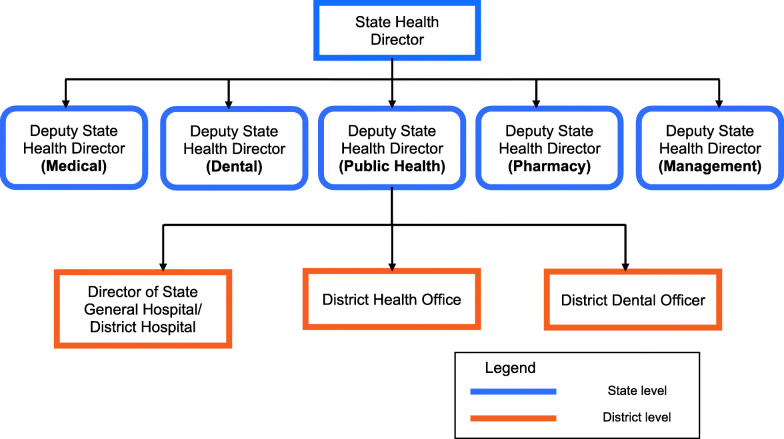
Organisation of State Health Department in relation to district-level state

Malaysia is situated in Southeast Asia; it is a former British colony with various inherited legacies, including the Malaysian health system, which is modelled after the National Health Service of the United Kingdom [26]. This study reports the qualitative component of a mixed-methods study conducted in the district of Gombak, situated in the state of Selangor. The other component of the concurrent mixed-methods research is a cross-sectional study that was conducted among healthcare personnel at the district level to examine their knowledge of, attitude towards and practices in relation to dengue prevention activities. The qualitative interviews were conducted with key informants in the districts of Klang, Petaling and Gombak until the point of saturation. The state of Selangor has nine district health offices, and the Gombak district health office has one of the highest burdens of dengue disease among all districts in Selangor. In Malaysia, the health system is imperially structured from the national and state levels to the local level, with relationships interlocked and dominated by the fraternity of the elite with well-organised special interests, whether direct or oblique. For this study, elite healthcare personnel were recruited from the Public Health Division of the Selangor State Health Department (Fig. 1). Most of the elites were stationed at Petaling and Klang. Elites working at public health divisions other than Petaling and Klang but relevant to the study were also recruited.

### Study outcomes

The primary objective of this study was to explore the predicament of governance in dengue prevention and control along with possible solutions. In part, this study also explored the methodological approach of a novice interviewing the elite.

### Study design and stakeholder selection

**Fig. 2:**
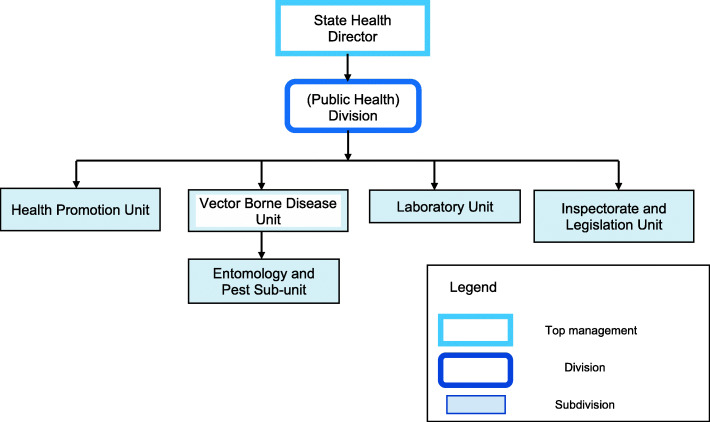
Organisation of State Public Health Division in relation to Vector-Borne Disease Unit

The qualitative study used a case-study approach. We aimed to interview elite healthcare personnel individually, face-to-face, to obtain their views on the governance of dengue prevention and control. Purposive sampling of elite healthcare personnel (State Health Department) was conducted based on selected domain expertise. The respondent(s) were selected based on purposive sampling with a minimum of one healthcare representative from each unit, as shown in Fig. 2. We aimed to interview at least one senior member of the unit and/or the head of unit from each unit or subunit. We sought the head of the unit first, and if she/he was unavailable, we reverted to the next in line or deputy of that unit or subunit. If new information was still being uncovered despite having interviewed both the head of the unit and his/her deputy, we continued to other respondents in the same unit until the point of saturation was reached, after which no new information was gathered from the interviewee. In this study, the saturation point was reached after the 19th interview.

### Content of and conduct of interviews

A semi-structured format was used for the interviews (Additional File 1), guided by an interview protocol that included questions on four principles of governance, i.e., accountability, rule of law, personnel performance, and stakeholder relations. The protocol was developed in English and Malay by RAM and NTR, who are both bilingual. Both languages are widely spoken in Malaysia. We pre-tested the interview protocol with five healthcare workers of different categories to check for face validity. Further improvements were made following the comments received during the pre-test.

Prior to conducting interviews with the elites, we performed groundwork, as we anticipated challenges from the elites of our knowledge and relevance to the subject matter, as reported by Harvey et al. (2015). There were instances when we found ourselves being asked questions and assessed by the interviewee. Hence, it was important to project a positive impression, constantly gauge the mise-en-scène of the interview and adjust behaviour, intonation, and mannerisms accordingly to gain the interviewees’ respect. This was important not only to gain respect but also to increase the likelihood of elite members providing access to other interview opportunities through personal recommendations [22, 27, 28].

NTR conducted all of the interviews between January 2019 and November 2019. They lasted 32–68 min. The interviews were conducted face-to-face at a location designated by the interviewee or the interviewee’s workplace, depending on the interviewee’s preference. The interviews were digitally recorded and transcribed verbatim. To facilitate unvarnished discussion, the interviewees’ confidentiality was assured by obtaining their consent.

### Ethical approval

This study was approved by the Ethics Committee for Research Involving Human Subjects of Universiti Putra Malaysia (JKEUPM), the Medical Research and Ethics Committee of Ministry of Health (MOH) and the Selangor State Health Department Quality Unit. Written consent was obtained from the participants. The names of the respondents were not disclosed in the study, and the audio recordings were saved on a password-protected computer.

### Data analysis

All qualitative data collected were analysed using NVivo version 11. We conducted a deductive thematic analysis aimed at identifying the themes associated with principles of good governance derived from the expressed narrative. The interviews were conducted mostly in English and Malay, which is the official language of Malaysia. Field notes were made by NTR promptly after the interviews. However, some of the audio-recorded interviews contained code mixing and/or code switching. Accounting for the latter issue presented a unique challenge, and the audio recordings had to be listened to several times before they fit the verbatim transcriptions compiled by NTR. The transcripts were carefully translated into English by NTR, and the forward-backward translation method was applied to five of the transcripts (20%) to check the accuracy of the data. The researchers studied the translations repeatedly to familiarise themselves with the data.

RAM then reviewed the translated transcripts of the interviews. Manual coding was performed by NTR and RAM. Codes that emerged from the first few translated interviews formed the basis of the codebook that was used to assess the subsequent translated transcripts. The initial coding process was expanded into focused coding. In focused coding, the association between different initial codes was explored on the basis of frequency, sequence, correspondence, and similarity. The other investigator (SRS) independently repeated this process iteratively for all of the transcripts based on the codebook. RAM and SRS then discussed the focused coding choices in detail. The final deductive codes were grouped into meaningful categories. AM assisted in reviewing and proofreading the transcripts. The sub-themes were generated by blending several categories together under the guidance of the main principles of existing governance theory, namely, corporate governance theory. The research team held regular meetings and discussions to cross-check the generated categories based on the objectives of the study. In case of any conflict or disagreement, the final decision rested upon RAM, who is a senior academician with vast experience in mixed-methods research.

### Patient and public involvement

Patients and the public were not involved in the planning or conduct of this study.

### Limits and biases

This study did not examine factors among the elites involved in dengue prevention and control activities who were not employed by the Ministry of Health; hence, the exclusion of many stakeholders was necessary to weave an inclusive framework of good governance.

While many of the elites interviewed for this study were willing to share their experiences and reflections, some of the interviewees appeared hesitant and had reservations about expressing their thoughts, especially those related to accountability, operational effectiveness and discrepancies related to dengue prevention and control.

## Results

### Participant characteristics

A total of 19 elite healthcare personnel were interviewed (Table 1). Five themes were developed from the categories on the current situation of the governance predicament of dengue prevention and control activities at district health offices. Quotations were used to illustrate and support points, with the position of the quoted elite described in brackets after each quotation in the corresponding tables. To preserve the anonymity of the respondents, some minor details were subjected to nondisclosure.

**Table 1:** Respondent characteristics and interview duration

Stakeholder	Respondent	Gender	Duration of interview (minutes)
**Vector-Borne Disease Unit**			
Senior Assistant Principal Director	1	M	65
Assistant Principal Director	2	M	47
Assistant Environmental Health Officer	3	F	55
Assistant Environmental Health Officer	4	F	58
Assistant Environmental Health Officer	5	M	36
Assistant Environmental Health Officer	6	F	49
**Inspectorate and Legislative Unit**			
Assistant Environmental Health Officer	1	M	53
Assistant Principal Director	2	F	44
Assistant Environmental Health Officer	3	M	39
Senior Assistant Environmental Health Officer	4	M	54
**Entomology and Pest Unit**			
Entomologist Science Officer	1	M	68
Entomologist Science Officer	2	F	62
Entomologist Science Officer	3	M	34
**Health Promotion Unit**			
Assistant Environmental Health Officer	1	F	45
Health Promotion Assistant Principal Director	2	M	55
**Senior Management**			
Assistant Principal Director	1	M	48
Chief Nurse	2	M	32
Assistant Environmental Health Officer	3	F	33
Senior Assistant Medical Officer	4	M	46

### Theme 1: public service accountability

There was wide agreement among the interviewees on the role of accountability as an important cornerstone of good governance. However, the context of the relationship between healthcare personnel and their community is anfractuous, i.e., the extent to which civil servants are answerable for their actions. One elite spoke at length on the concept of public service accountability, which encompasses the organisational structure, legal and reporting framework, strategy, and procedures. Another noted that any organisation that uses public money and makes decisions needs to be accountable for decisions made, especially those that have an impact on the livelihood of a community (Table 2).

**Table 2:** Public service accountability

Theme	Categories	Codes	Quotations
Public Service Accountability	Role and Responsibility of Decision Makers	Doing it right, integrity and its challenges, adherence to collective responsibility,	I try my level best to make the best decision based on my available resources [frowns]. … There are of course consequences, I know … but being accountable, yes … I mean how? It is a collective decision most of the time, and in many circumstances, I go with the general consensus … but most of the time, we carry out orders based on existing standard operating procedures (SOPs). (Entomologist 1)
	Steadfast Leadership	Time-based promotion, meritocracy ineptitude in public service, conventional mindset	I think there are good-quality leaders …. at the district level, here in the state department [taps left index finger at table]. … Now, for this staff to become effective … if they are to be effective, public sector leaders need sufficient freedom to lead and to be supported and challenged by others within and beyond their organisations. … The problem is [pause] … there is not much challenge from within …. [slight rise of intonation] [ends with chuckle]. ...So yes, if you ask me, steadfast … yes. (Inspectorate and Legislative Unit 3)
	Underlying Hindrances and Constraints	Handling mounting public expectations, the conundrum of intermediaries, serving various stakeholders	From my understanding … to have good governance, you need healthcare staff that have good characteristics. … Forget about the system; look at the people [healthcare personnel]. Us … [mid-level healthcare personnel], we don’t decide on the policies; we act as intermediaries between the district and Putrajaya. … Sometimes people in the district, they don’t understand, and they blame us. … They get unhappy when their request, applications are not met … and the programme is not conducted in the way it is supposed to be? [questions the interviewer] (Inspectorate and Legislative Unit 1)

The elites described why decision makers in leadership positions should also prioritise succession planning via the identification of potential talent as an embodiment of accountability. The development of career pathways to retain leaders in the public sector reflects efforts towards sustainable and steadfast leadership. By general consensus, for effective governance to materialise, a majority of the elite felt that devolving administrative responsibilities via effective decentralisation would lead to better accountability practices, especially in the district health offices, by doing away with unnecessary constraints.

### Theme 2: effectiveness and efficiency

Being efficient and effective are key features of good governance, with the local health district in essence compelled to find ways to make the best use of available resources (human, material, financial, etc.) [12]. The process is gradually overwhelmed by inherent determinants such as urbanisation that are beyond the district health office jurisdiction. The elites also commented that rising public expectations in regard to public sector performance require healthcare personnel in public service who are able to integrate and embrace a wider set of skills while maintaining the antecedent strengths of public sector standards and non-profit fundamentals. Hence, to maintain traction in regard to public expectations, the elites felt that innovation is essential to all aspects of public health strategy and programme development. The interviewees also identified the need to develop evidence-based mechanisms to increase the chances of success for implemented activities. However, three elites expressed frustration regarding the lack of necessary characteristics or skills demonstrated by rank-and-file personnel (Table 3).

**Table 3:** Effectiveness and efficiency

Theme	Categories	Codes	Quotations
Effectiveness and Efficiency	Capacity for delivering performance	Need for strategic planning, setting aside differences and indifference	We can’t stop urbanisation or people coming in and putting up homes in Selangor; those are beyond our jurisdiction [pause] … but what we can control and improve are the capacities of our human resources, logistics, joint efforts among our own units. … In the smaller districts, I see more cooperation … but in the bigger districts, it is more difficult to put aside the differences and work together … too many agendas [laughs]. (Senior Management 2)
	Healthcare personnel development	Trimming excesses, empowering workers, transparency in the workflow process, listening with empathy, engaging the right instrument for the work	We can allocate more funding to rent more vehicles used for fogging activities … hire more temporary workers … purchase more chemicals for vector control … but is it effective or efficient when the healthcare personnel that implement or take part in the prevention and control activities … [pause] [lowers head] … lack the necessary characteristics or skills?...So you must made sure they [decision makers at the district level] are well trained and motivated as well. … The chain of command must be developed to ensure that resources are mobilised to deliver good services to the community … . (Inspectorate and Legislative Unit 3)
	The need for leadership to spearhead the programme	Differences between leadership and management, micro-management versus macro-management, the ability to direct and navigate	Officers in charge of vectors must be able to connect the dots to see the big picture … to prevent an outbreak from progressing to *hotspot* status. They must be able to understand the factors and deal with them … or correctly pinpoint the problem …. But what I see is … a vector officer gets too immersed in the day-to-day unimportant matters. … Leave those non-urgent matters to your U32 or U29. … Put more focus on the bigger and more important things …. To stay effective … we need to be able to step back … .to focus on the bigger picture … get it [emphasise]. (Vector-Borne Disease Unit 3)

The elites widely agreed that for good governance to thrive at the district health offices, the well-being of healthcare personnel needs to be prioritised and safeguarded. Most, if not all, healthcare personnel are resilient and dedicated in their pursuit of providing high-quality service; however, operating in a less-than-optimal environment can gradually undermine their performance. The interviewees felt that this problem could be solved by competent leaders spearheading programmes and filling governance training gaps.

### Theme 3: regulation and rule of law

Eight of the elites mentioned that regulatory constraints were a perceived governance impediment. Without a proper regulatory framework, the establishment of trust, flexibility of work processes and stability needed for medium- and long-term planning in dengue prevention and control programmes are likely to deteriorate, according to the interviewees. However, facilitating harmony among the various stakeholders and promoting governance in the regulatory ecosystem are challenges in themselves, according to the elites. One of the interviewees described the multi-faceted layers of regulation and work procedures at the district health offices, ranging from active to passive surveillance to offsite and onsite enforcement of regulations (Table 4).

**Table 4:** Regulation and rule of law

Theme	Categories	Codes	Quotations
Regulation and Rule of law	Healthcare personnel compliance with regulations	Setting the tone, developing the right strategy, human resources and capacity management	Ideally … we would like to ensure that all outbreaks do not progress and become uncontrolled outbreaks and … hotspots … ideally [pause]. … So in the bigger districts … we have to choose our battles [outbreak localities] wisely to ensure that our staff, logistics, and financial resources are well utilised … setting aside the compliance issue. (Senior Management 2)
	Regulatory clarity	Operating in grey areas, fiduciary concerns, adherence to public accounting framework	The aspect of budgeting and money availability is very important and often overlooked in public health programmes …. I give you examples [pause] [starts listing on paper] … the Hilux [pickup truck] rental … maintenance of equipment for fogging activities … the payment of temporary workers …. money to organise workshops and courses for the staff to improve their dengue skills …. It takes a lot of money to run these [dengue] programmes. (Vector-Borne Disease Unit 6)
	A conducive regulatory ecosystem	Enforcement inadequacy, humanising the law, adopting a holistic approach	If you ask me …. I think the existing law is good enough, but we need to consider many other factors … before coming out with compounds …. Whether the individual can pay … his housing condition … his environment …. When you hand out compounds … you punish the offender … but you also make it difficult for the vector team to go into that community again in the future …. They don’t trust us; they close their doors …. they act like they are not at home …. [laughs] (Inspectorate and Legislative Unit 4)

More than half of the elites identified the lack of a good governance framework for healthcare personnel in the course of dengue prevention and control engagement with the communities. One of the interviewees bluntly remarked that counterproductive actions such as issuing summonses or compounds in a community would likely backfire in terms of cultivating goodwill, since the law is discriminatory when applied injudiciously.

In summary, the majority of the elites felt that for good governance to expand at the district health office level, healthcare personnel need to be refreshed on how laws and regulations function at the point of their initiation into civil service and every so often afterwards. Healthcare personnel should be reminded of the external consequences of infringement when engaging with the community in dengue prevention and control activities, the internal consequences of violating the code of practice, and the risk of being reprimanded by the department’s whip for noncompliance with protocols.

### Theme 4: community participation as stakeholders

A recurring theme of the elite supported the assumption that positive reciprocity from the community can no longer be expected when public policies are delivered merely as an afterthought. Most of the participants noted that public health programmes delivered successfully at the district level require good governance, co-production and involvement and rapport with the community initiated by healthcare personnel.

The majority of the interviewees felt that for good governance to expand to district health offices, community participation was essential. However, the elites identified that first, the underlying needs and concern of the community must be identified, and when these levels have been determined, then specific interventions can be tailored in accordance with the local needs and requirements of the community (Table 5).

**Table 5:** Community participation as stakeholders

Theme	Categories	Codes	Quotations
Community Participation as Stakeholders	Community mobilisation and empowerment	Listening to grassroots, policy implementation challenges, shifting responsibility to the community	If you ask me … I personally think the vector staff needs to do more health education campaigns … to increase the awareness of recommended vector control practices … like spending time checking for breeding at your own house … and cultivating more active participation by community members in reducing Aedes … and of course the staff [healthcare personnel] needs to be more innovative and proactive … [slight rise of intonation] during … the prevention and control activities on the ground. … (Vector-Borne Disease Unit 2)
	Cultivating the trust of the community	Acculturation of new policies, building connections with the community, matters of the heart	The current implementation of the dengue ecosystem... [pauses and explains], which is a brainchild of our last TPK for Public Health, brings the healthcare personnel closer to the community … especially the troublesome ones …. The vector team goes into the area even when there is no outbreak … no cases …. The idea of the ecosystem … simply say … is to assess the locality, create a profile of potential breeding sites …. It is more of a preventive job …. During this ‘walkabout’ by our staff while gathering data … they can get to know the people staying there as well. … (Inspectorate and Legislative Unit 2)

### Theme 5: collaboration and engagement

The elites agreed that coordination and diffusion of responsibility for public health initiatives throughout government and society can be improved with genuine collaboration. One of the participants stated that health has become critical macro-economic and political fodder for certain actors in the current state of affairs, especially when governments, communities and citizens are increasingly engaged in governance for health, whether as state or nonstate stakeholders (Table 6).

**Table 6:** Collaboration and Engagement

Theme	Categories	Codes	Quotations
Collaboration and Engagement	Forging mutually dependent partnerships	A climate of distrust, paternalistic legacies, managing prejudice, working towards a mutual objective	Nowadays … there seems to be a lot of talk about silo this … silo that, about breaking down silos … let’s look at our own [emphasise] organisation first....Are we ready to work together in an open way with other agencies? Why do you think at some districts, the private PCO did not last long? … Of course there are many reasons … if you start pointing fingers at each other....[laughs] (Inspectorate and Legislative Unit 3)
	Beyond the domain of the health system	Stretching the limits, the rapid pace of progress, insufficient capabilities and capacities	I think we are quite aware … that contributing factors like rapid urbanisation, poor garbage disposal and basic sanitation, increased mobility. … I mean movement of people, which is getting further and wider … foreigners staying at apartments and flats housing … made it more difficult to cut out dengue …. You get what I mean. … It is not that straightforward … . (Entomologist 2) To bring dengue cases under control … I am talking about long-term measures here … will require strong partnership between local authorities [district health offices] and local communities … and also other local stakeholders … be it the NGOs, local businesses … concerned individuals or champions … that have an active interest …. [P15] (Assistant Environmental Health Officer)

The majority of the elites felt that for good governance to expand at the district health office level, the underlying complexity of the dengue prevention and control programme needs to be acknowledged and particularised. Notwithstanding their frustration, the interviewees sensed that the multifarious complexity necessitates decision makers and stakeholders clarifying and collaborating with better-informed entities regarding how to implement a strategic framework for healthcare personnel to control and prevent dengue outbreaks.

## Discussion

Governance is about power, relationships, and accountability—who has influence, who decides, how citizens and other stakeholders have their say, and how decision makers are held accountable [29]. In this study, elite or rank-and-file healthcare personnel who intentionally underperform have led to many problems at the local district health level. Hence, enhancing accountability in health systems through the use of various check-and-balance mechanisms is much emphasised and crucial for improving the governance and quality of the health services delivered. Studies reported by Beh (2011), Smith et al. (2012) and Bao et al. (2013) discussed the establishment of fair and impartial human resources management practices that are essential for promoting good governance among public servants [2, 12, 30]. However, if public servants perceive that they are underpaid, overworked and lack job security, they are less likely to embrace initiatives to improve performance and become effective or efficient [2, 12, 26].

In this study, the elites agreed that the state health department and health district offices continuously endeavour to galvanise and improve the capacities of dengue prevention and control programmes among healthcare personnel. Nonetheless, the management of public health activities is particularly challenging because, unlike in the private sector, where feedback on performance is demonstrated via clear metrics, the performance and impact of public health programmes such as dengue prevention and control activities are very often difficult to track in real time [29]. For healthcare personnel to comply with good governance, prerequisite conditions, such as transparency, clear promotion stages and trust in top and middle management, are decisive for establishing a performance-oriented culture [10]. The absence of these attributes may result in poor health system performance due to corruption, nepotism, lack of transparency, political interference and inadequate use of information [31]. Based on these findings, a conducive climate that will promote non-punitive actions by top and middle management will translate into a performance-oriented culture among rank-and-file healthcare personnel. The submission to feudalism culture that is prevalent in the health system has hindered constructive and progressive actions in the past and clogged the machinery of good governance.

Most of the elites identified the need to enhance the current regulatory framework for healthcare personnel. A fortified rule of law will contribute to the establishment of trust, flexibility of the work process and stability needed for medium- and long-term planning in the dengue prevention and control programme. However, the archaic work process does not bode well for the changing landscape, in which the general public demands more accountability from public servants and healthcare personnel [32]. Hence, the management of personnel needs to be appropriately institutionalised via a framework of law to ensure the realisation of several principles of governance (transparency, rule of law, accountability, etc.) that in absentia could undermine the public service value system. The need for a framework has been emphasised by Palagyi et al. (2019) as a building block of good governance in ensuring a resilient and strong healthcare system to prepare countries for the threat of emerging infectious diseases [16].

The elites frequently spoke about how public health services at the local district level should also leverage existing key units, i.e., surveillance, entomology, and enforcement in other agencies, for better decision making and efficient use of resources. In addition, better support from relevant agencies for public health advocacy can be realised if public health and urban planners collaborate in the creation of a non-conducive environment that can hamper potential vector breeding sites. This study also suggests that wider engagement with other agencies and groups, i.e., district education offices, local law and enforcement units, and the corresponding municipal councils of Gombak District, could be beneficial.

To pool resources, there needs to be a more participatory approach at the local level pivoting around common interests. Hence, healthcare personnel who are key decision makers need to forge partnerships with community leaders to generate more constructive communication and collaboration by empowering them to participate in dengue control and prevention activities [33]. A well-planned intervention programme that includes community governance has resulted in better community knowledge, attitudes and practices in dengue prevention; increased household and community participation; and improved the involvement of stakeholders with prospects for sustainability [34].

There were a number of challenges in interviewing people in positions of power, such as the interviewer-interviewee relationship, gaining access and unnecessary distraction. Elites are used to taking the initiative, issuing directions, leading strategic planning, and asserting themselves in relation to those with whom they work. Such a demeanour can lead to an imbalance between a novice interviewer and an assertive interviewee, resulting in the interviewer being unable to be parrhesiastic in his conduct. This issue of power is a significant determinant in interviewing elites and has attracted a substantial literature [21, 27, 28].

The process of gaining access to the elites occurred largely through referrals. In this context, starting the interviews with ultra-elites from the eagle’s perch facilitated unceremonious access to other elites via referrals. Setting the appointments was a challenge, but patience and perseverance were rewarded once the elites agreed to be interviewed. To prevent distraction, the designated location for conducting interviews should be situated away from the elite office or workstation. This strategy will lessen interruptions from unannounced walk-ins by personnel seeking engagement with the interviewee for various reasons.

## Conclusion

Political arrangements at the national level will establish the tone of how governance practice will be operationalised by entities and individuals at lower levels of the health system. Giving too much attention to the infallibility of the elite risks side-lining the expression of rank-and-file personnel. Based on the current intergovernmental structure that constitutes federalism, the trajectory is unlikely to deviate, as are the predicaments. This study advocates the development of a good governance framework for the dengue prevention and control programme that integrates the core principles of good governance across multiple levels of healthcare delivery. The framework will serve as a guideline and epitomise a model for other districts where the burden of dengue places significant constraints on healthcare personnel to deliver health services in tandem with good governance.

## Supplementary Information

**Additional file 1.** Semi Structured Interview Guide – the list of questions and probes used during the interview.

## Data Availability

Anonymous interview transcripts will be shared upon receiving reasonable request. Please contact the corresponding author through this email, rosliza_abmanaf@upm.edu.my.

## Abbreviations

ANAO: Australia national audit office
AM: Aidalina mahmud
NTR: Ng tyng ruey
RAM: Rosliza abdul manaf
SRS: Siti rohana saad

## References

[1] Nanoot M, Weerasak PN. Global health disruptors: the rise of civil society. 2018; Available from: https://blogs.bmj.com/bmj/2018/11/28/global-health-disruptors-the-rise-of-civil-society/. Accessed 22 July 2017.

[2] Beh L-S. Formulating good governance and moving forward: navigating transparency or reinforcing authority and Power_ 2011.

[3] Liese B, Rosenberg M, Schratz A. Programmes, partnerships, and governance for elimination and control of neglected tropical diseases. Lancet 2010;375(9708):67–76. Available from: http://dx.doi.org/10.1016/S0140-6736(09)61749-9 10.1016/S0140-6736(09)61749-9 20109865

[4] Abimbola S, Negin J, Jan S, Martiniuk A (2014). Towards people-centred health systems: a multi-level framework for analysing primary health care governance in low- and middle-income countries. Health Policy Plan.

[5] Weintraub RL, Wachter K, Goldsmith J, Teichman MJ, Algur E, Rosenberg JD. Assessment of management capacity to improve the value of health-care systems: a survey. Lancet Glob Health. 2017;5:S10 Available from: http://linkinghub.elsevier.com/retrieve/pii/S2214109X17301171.

[6] Chongsuvivatwong V, Phua KH, Yap MT, Pocock NS, Hashim JH, Chhem R, Wilopo SA, Lopez AD (2011). Health and health-care systems in Southeast Asia: diversity and transitions. Lancet.

[7] Kickbusch I, Gleicher D. Governance for health in the 21st century. Who 2012;1–106.

[8] Koh T. The Principles of Good Governance. Bangkok; 2009. Available from: https://lkyspp.nus.edu.sg/docs/default-source/gia-documents/sp_tk_the-principles-of-good-governance_[accessed 22 Jul 2017].

[9] Maire S, Bouchard J. rethinking operational governance the nervous system that ensures the company is both flexible and resilient . 2015. Available from: https://www.oliverwyman.com/content/dam/oliver-wyman/global/en/2015/dec/Rethinking-Operational-Governance-2016.pdf [accessed 3 Jul 2019].

[10] Caroline J. Good governance and government sector performance at Majlis Perbandaran Seberang Perai. 2015;(January):1–121.

[11] Montes MF, Reyes RA, Tabunlertchai S. Macroeconomic management in Southeast Asia’s transitional economies. 1995; Available from: http://scholarspace.manoa.hawaii.edu/handle/10125/23842 (accessed 15 Mar 2019).

[12] Smith PC, Anell A, Busse R, Crivelli L, Healy J, Lindahl AK, Westert G, Kene T (2012). Leadership and governance in seven developed health systems. Health Policy (New York).

[13] Klomp J, De Haan J (2008). Effects of governance on health: a cross-national analysis of 101 countries. Kyklos.

[14] Ciccone DK, Vian T, Maurer L, Bradley EH (2014). Linking governance mechanisms to health outcomes: a review of the literature in low-and middle-income countries. Soc Sci Med.

[15] Petersen I, Marais D, Abdulmalik J, Ahuja S, Alem A, Chisholm D, Egbe C, Gureje O, Hanlon C, Lund C, Shidhaye R (2017). Strengthening mental health system governance in six low-and middle-income countries in Africa and South Asia: challenges, needs and potential strategies. Health Policy Plan.

[16] Palagyi A, Marais BJ, Abimbola S, Topp SM, McBryde ES, Negin J (2019). Health system preparedness for emerging infectious diseases: a synthesis of the literature. Glob Public Health.

[17] Bismark MM, Studdert DM (2014). Governance of quality of care: a qualitative study of health service boards in Victoria, Australia. BMJ Qual Saf.

[18] Atif M, Malik I, Mushtaq I, Asghar S (2019). Medicines shortages in Pakistan: a qualitative study to explore current situation, reasons and possible solutions to overcome the barriers. BMJ Open.

[19] Siddiqi S, Masud TI, Nishtar S, Peters DH, Sabri B, Bile KM (2009). Framework for assessing governance of the health system in developing countries: gateway to good governance. Health Policy (New York).

[20] Harvey A. ORE Open Research Exeter Title Strategies for conducting elite interviews a note on versions . 2015. Available from: http://hdl.handle.net/10871/16200 (assessed 5 Feb 2018).

[21] Conti JA, O’Neil M. Studying power: qualitative methods and the global elite. Qual Res. 2007;7(1):63–82. [cited 2020 Apr 24] Available from: http://journals.sagepub.com/doi/10.1177/1468794107071421

[22] Liu X. Interviewing elites: Methodological issues confronting a novice. Int J Qual Methods. 2018;17(1):1609406918770323.

[23] Mahathir M. A doctor in the house: the memoirs of Tun Dr. Mahathir Mohamad by Mahathir bin Mohamad 2011: 301–311.

[24] Mudin RN (2015). Dengue incidence and the prevention and control program in Malaysia. Int Med J Malaysia.

[25] Ministry of Home Affairs Malaysia. Official Documents. National Publication,. 2011.

[26] MHSR. Malaysia Health Systems Research Volume I. 2016 [cited 2017 Dec 27]; Available from: http://www.moh.gov.my/penerbitan/Laporan/Vol 1_MHSR Contextual Analysis_2016.pdf.

[27] Stephens N (2007). Collecting data from elites and ultra elites: telephone and face-to-face interviews with macroeconomists. Qual Res.

[28] Mikecz R (2012). Interviewing Elites. Qual Inquiry.

[29] Ellen K, Robert H B. The Palgrave International Handbook of Healthcare Policy and Governance. 2015 [cited 2018 May 17]; Available from: http://www.ciando.com/img/books/extract/113738493X_lp.pdf

[30] Bao G, Wang X, Larsen GL, Morgan DF (2013). Beyond new public governance: a value-based global framework for performance management, governance, and leadership. Adm Soc.

[31] Sitienei JC, Nangami M, Manderson L, Paina L, Kosgei R (2017). Decision makers’ perspectives on implementation of governance attributes in the Kenyan Department of Health: a qualitative review in Uasin Gishu County. Lancet Glob Heal.

[32] Jaafar S, Noh KMMK (2012). Malaysia health system review. Health Syst Trans.

[33] Chang MS, Christophel EM, Gopinath D, Abdur RM (2011). Challenges and future perspective for dengue vector control in the Western Pacific region. West Pac Surveill Response.

[34] Tana S, Umniyati S, Petzold M, Kroeger A, Sommerfeld J (2012). Building and analyzing an innovative community-centered dengue-ecosystem management intervention in Yogyakarta, Indonesia. Pathogens Glob Health.

