# Supplementary material for: A qualitative study of governance predicament on dengue prevention and control in Malaysia: the elite experience
Source: BMC Public Health. 2021 May 6;21:876. doi: 10.1186/s12889-021-10917-3 (PMC8101109; doi:10.1186/s12889-021-10917-3)
Supplement: Supplementary file 1 — Additional file 1. Semi Structured Interview Guide – the list of questions and probes used during the interview. [file 12889_2021_10917_MOESM1_ESM.pdf]

Semi Structured Interview Protocol

This Semi Structured Interview have five (5) sections:	
Section A	**Structural Governance - Accountability**
Section B	**Regulatory Governance – Control/Rule of Law**
Section C	**Operational Governance - Performance**
Section D	**Community Governance – Stakeholder Relations**
Section E	**Background information/Socio-demographic characteristics**

1) Qualitative interview description
    i. Length : 45 – 60 minutes
    ii. Primary Goal: Key informants (KI); your opinions and what you think or feel about the topics that will be covered.

2) Consent:
    i. Written consent
    ii. Permission to have interview recorded

Section A : Structural Governance – Accountability

Main Questions
1) Can you describe on dengue prevention and control programme?<br>**Probe:** What is the role of Public Health Specialist/physician?<br>Who is involved? Who are the decision makers?
2) Could you describe the current collaborative efforts between sectors to effectively deal with dengue prevention and control?<br>**Probe:** What are the barriers towards collaborative efforts? How can we mitigate the issues?
3) Can you describe how effective is top-down approach in dengue outbreak operations?<br>**Probe:** How can we improved on the effectiveness?
4) In your opinion, what is governance?<br>**Probe:** What does good governance means to you? Why is governance important?

Section B: Regulatory Governance – Control/ Rule of Law

Main Questions
1) Can you describe the effectiveness of our legislation towards dengue threat?<br>**Probe:** Why are we not effective? How can we improve our legislation? What is lacking?
2) Can you describe on the accuracy and timeliness of reported events (dengue cases)?<br>**Probe:** Why is the data inaccurate? How can we improved on the reporting?

Section C: Operational Governance – Performance

Main Questions
1) Can you describe the capacity of a district to handle dengue outbreak? (e.g: human resource, facility,equipment)<br>**Probe:** What are the constraints/problems?, How can we address these issues?
2) In what ways can our surveillance system for dengue be improved?(online and offline)<br>**Probe:** What are the problems? How can we address them?
3) Can you described on our collaborative efforts with other relevant agencies in dengue prevention and control?<br>**Probe:** What are the concerns and constraints? How can we improve?

Section D: Community Governance – Stakeholder relations

Main Questions
1) Can you describe on COMBI programme in reducing dengue among participating communities?<br>**Probe:** What are the problems/shortcoming? How can we made COMBI more effective?How can we empower the community?
2) Can you describe on other health promotion programs to educate the public on measures to handle dengue disease?<br>**Probe:** How effective are these methods? Why is the uptake not encouraging?
3) Based on your experience, how can we better engage with the community?<br>**Probe:** How do we improved their awareness? What are the constraints?

Section E: Socio-demographic Characteristics

A1	*Age*	*Years of age (as of 1 January 2018)* -----------
A2	*Birthdate*	------------------------ DD/MM/YYYY (eg: 01/01/1980)
A3	*Gender*	☐ *Male*<br>☐ *Female*
A4	*Ethnicity*	☐ *Malay*<br>☐ *Chinese*<br>☐ *Indian*<br>☐ *others* ;______________________
A5	*Marital status*	☐ *Single*<br>☐ *Married*<br>☐ *Widowed/ Divorced*
A6	*Level of Education*	☐ *Pre- University*<br>☐ *University*<br>☐ *Postgraduate*<br>☐ *others;* ______________________
A7	*Employment Position*	Kindly state: ____________________
